# Supplementary figures and images for: Response to early drought stress and identification of QTLs controlling biomass production under drought in pearl millet
Source: PLoS One. 2018 Oct 25;13(10):e0201635. doi: 10.1371/journal.pone.0201635 (PMC6201870; doi:10.1371/journal.pone.0201635)

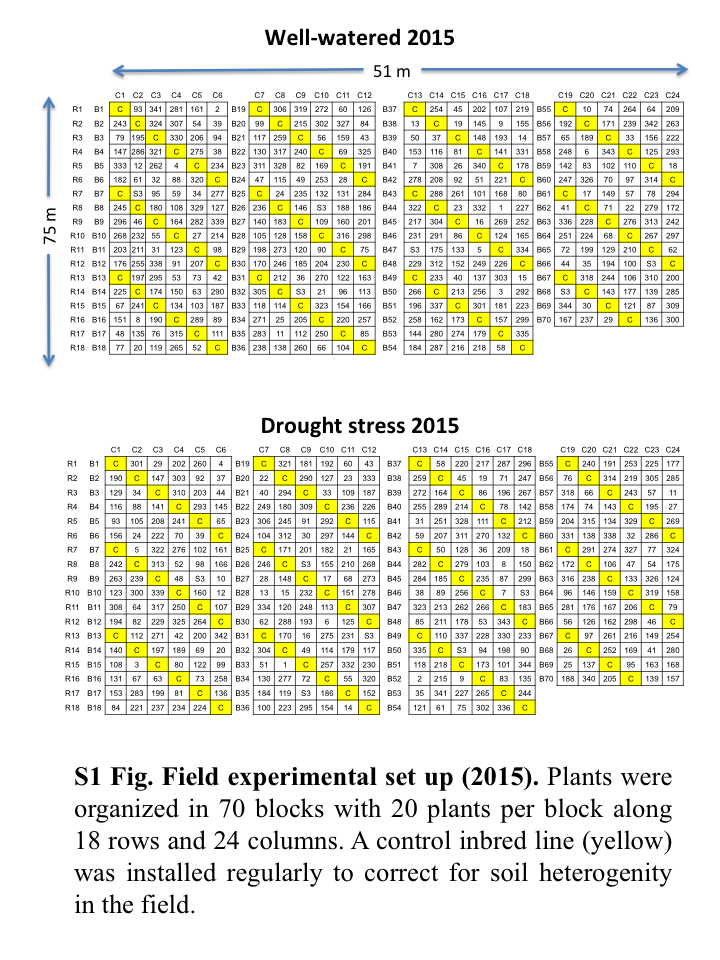

Supplement: S1 Fig — Plants were organized in 70 blocks with 20 plants per block along 18 rows and 24 columns. A control inbred line (yellow) was installed regularly to correct for soil heterogenity in the field. (TIFF) [file pone.0201635.s001.tiff]

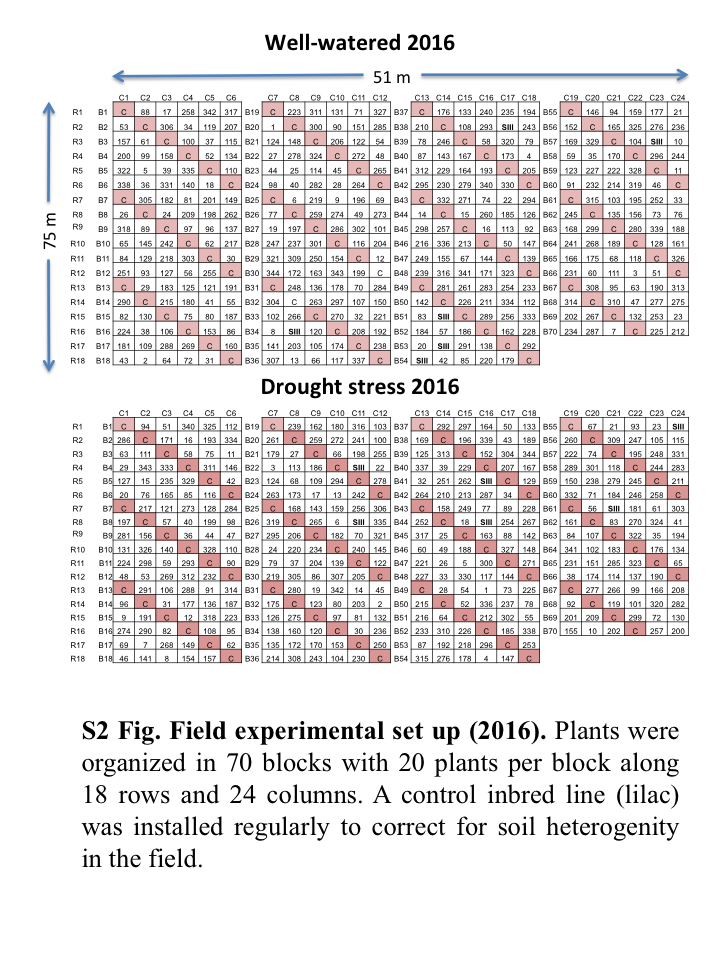

Supplement: S2 Fig — Plants were organized in 70 blocks with 20 plants per block along 18 rows and 24 columns. A control inbred line (yellow) was installed regularly to correct for soil heterogenity in the field. (TIFF) [file pone.0201635.s002.tiff]

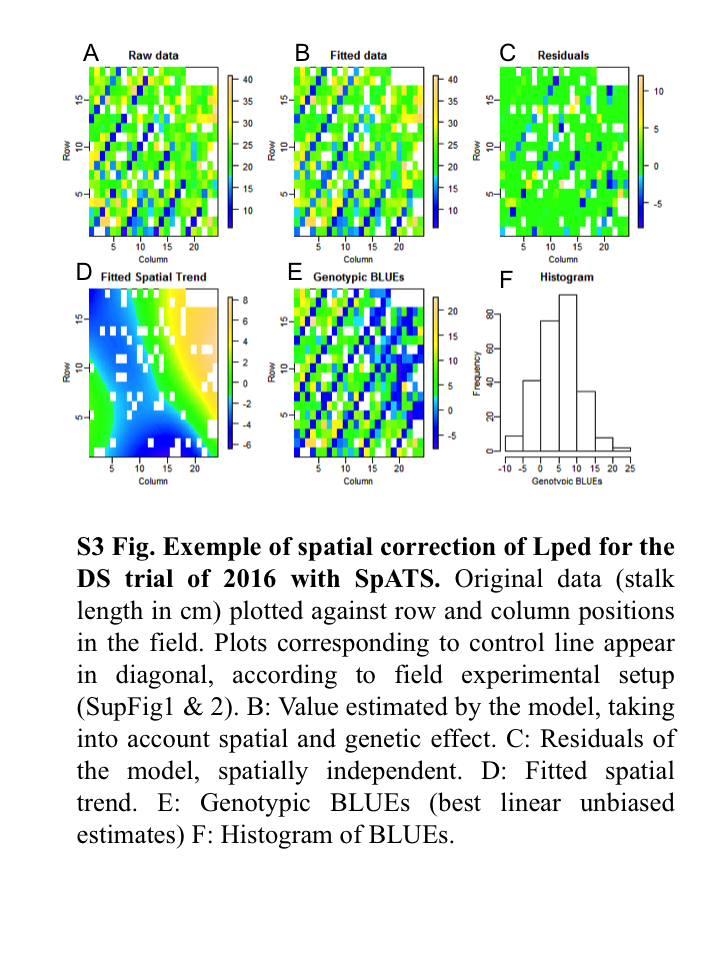

Supplement: S3 Fig — A: Original data (stalk length in cm) plotted against row and column positions in the field. Plots corresponding to control line appear in diagonal, according to field experimental setup (S1 and S2 Figs). B: Value estimated by the model, taking into account spatial and genetic effect. C: Residuals of the model, spatially independent. D: Fitted spatial trend. E: Genotypic BLUEs (best linear unbiased estimates) F: Histogram of BLUEs. (TIFF) [file pone.0201635.s003.tiff]

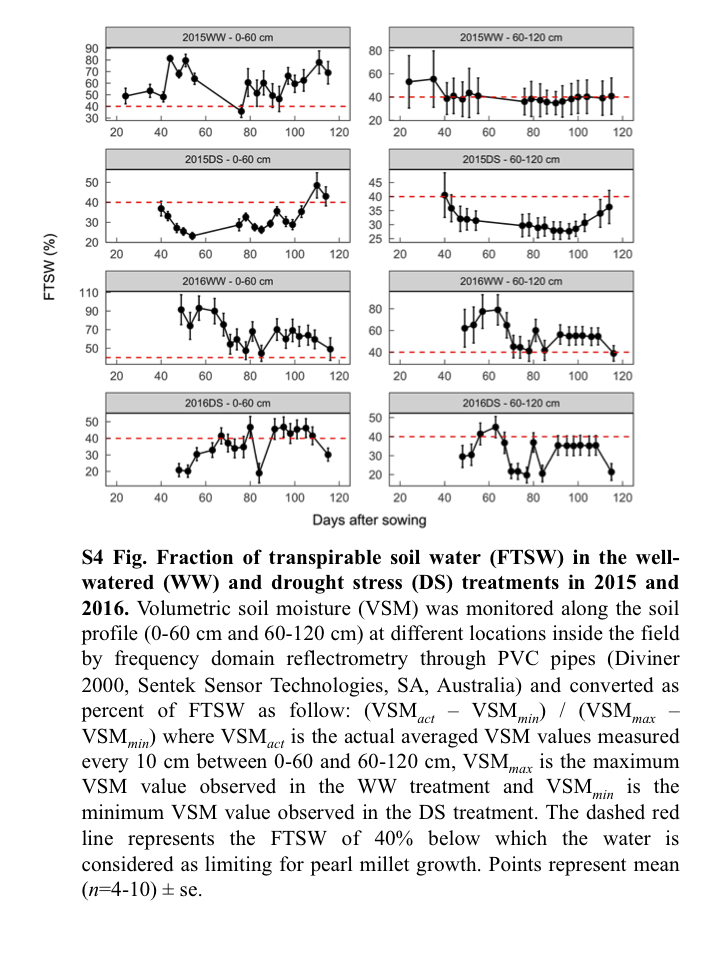

Supplement: S4 Fig — Volumetric soil moisture (VSM) was monitored along the soil profile (0–60 cm and 60–120 cm) at different locations inside the field by frequency domain reflectrometry through PVC pipes (Diviner 2000, Sentek Sensor Technologies, SA, Australia) and converted as percent of FTSW as follow: (VSMact–VSMmin) / (VSMmax–VSMmin) where VSMact is the actual averaged VSM values measured every 10 cm between 0–60 and 60–120 cm, VSMmax is the maximum VSM value observed in the WW treatment and VSMmin is the minimum VSM value observed in the DS treatment. The dashed red line represents the FTSW of 40% below which the water is considered as limiting for pearl millet growth. Points represent mean (n = 4–10) ± se. (TIFF) [file pone.0201635.s004.tiff]

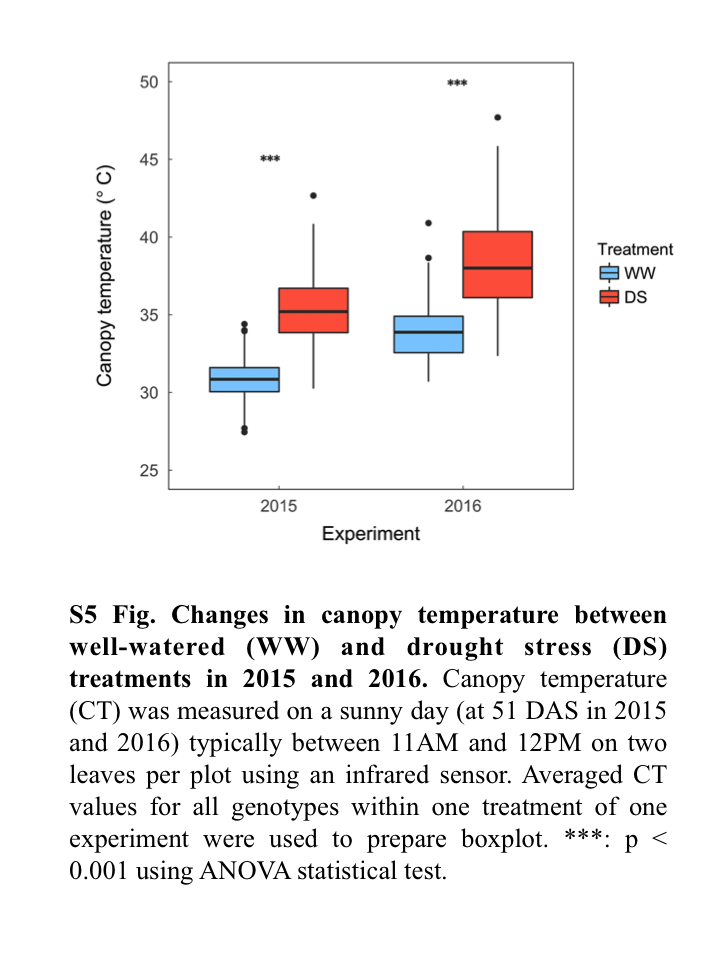

Supplement: S5 Fig — Canopy temperature (CT) was measured on a sunny day (at 51 DAS in 2015 and 2016) typically between 11AM and 12PM on two leaves per plot. Averaged CT values for all genotypes within one treatment of one experiment were used to prepare boxplot. ***: p-value < 0.001 using ANOVA statistical test. (TIFF) [file pone.0201635.s005.tiff]

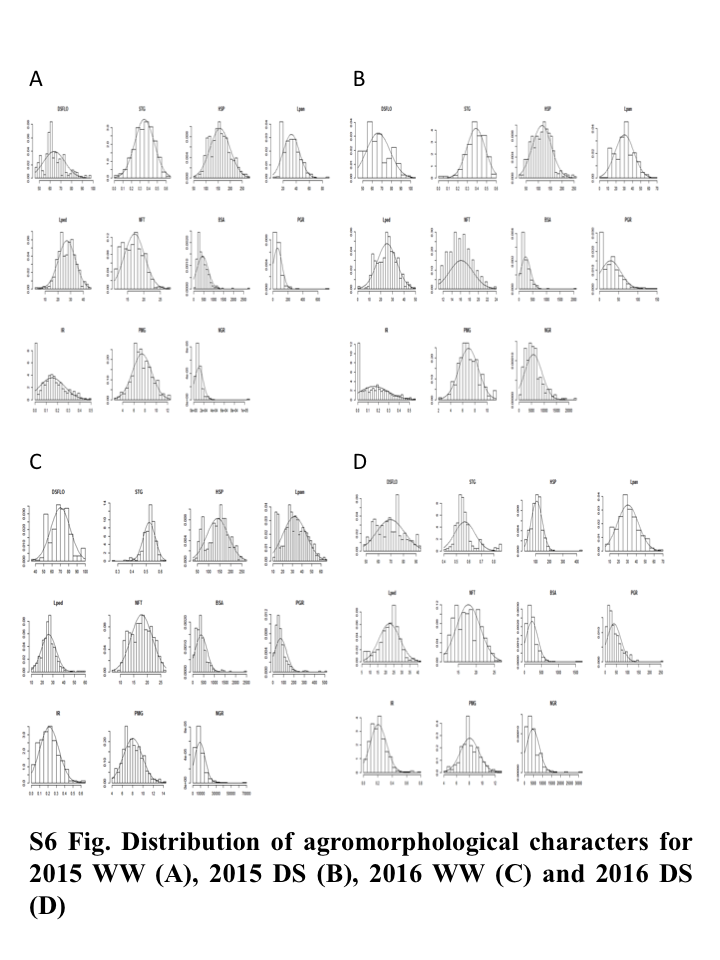

Supplement: S6 Fig — Distribution of agromorphological characters for 2015 WW (A), 2015 DS (B), 2016 WW (C) and 2016 DS (D). (TIFF) [file pone.0201635.s006.tiff]

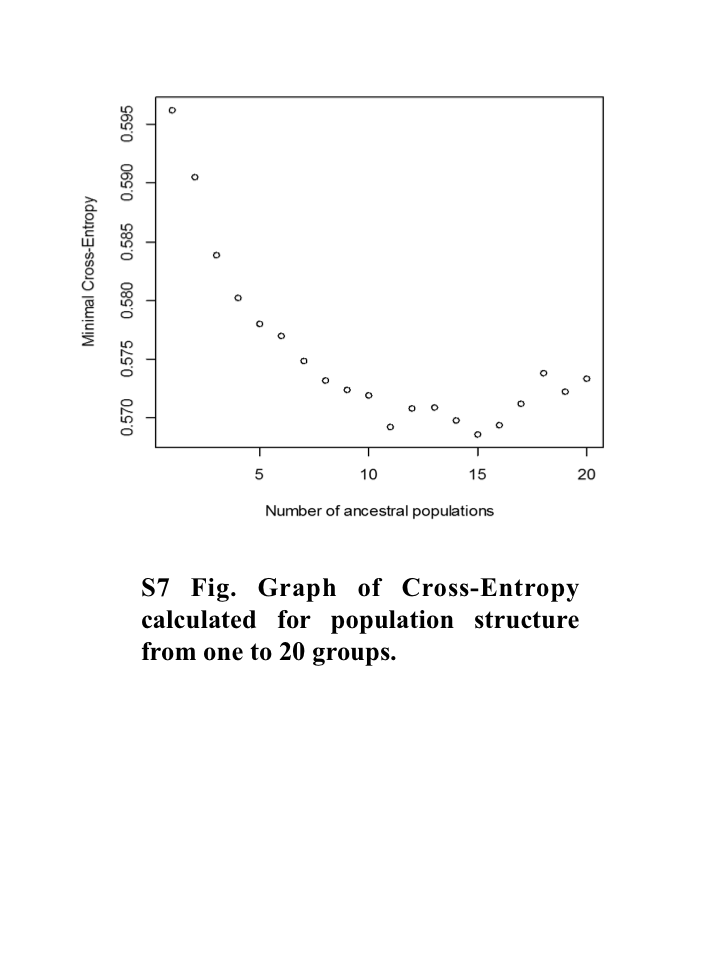

Supplement: S7 Fig — (TIFF) [file pone.0201635.s007.tiff]
